# Youth exposure to violence and victimization in a South African community sample

**DOI:** 10.4102/sajpsychiatry.v30i0.2311

**Published:** 2024-09-30

**Authors:** Lingum G. Pillay, Basil J. Pillay, Wilbert Sibanda

**Affiliations:** 1 Department of Behavioural Medicine, College of Health Sciences, University of KwaZulu-Natal, Durban, South Africa; 2 School of Mathematics, Statistics and Computer Science, College of Agriculture, Engineering and Science, University of KwaZulu-Natal, Durban, South Africa; 3 Department of Health Science Biostatistics, Faculty of Health Sciences, Nelson Mandela University, Port Elizabeth, South Africa

**Keywords:** youth, risk factors, sociodemographic risk, psychosocial risk, maternal education, violence exposure, peer victimisation, cyber victimisation, internalizing disorders

## Abstract

**Background:**

Studies show that youth in low socioeconomic communities suffer significant disturbances in mental and emotional health because of exposure to violence and peer victimisation, manifesting in internalising disorders such as depression, anxiety and traumatic stress.

**Aim:**

To examine the relation between risks and exposure to community violence and peer victimisation.

**Setting:**

Low socioeconomic communities in Durban, KwaZulu-Natal.

**Methods:**

Data were collected via school and home interviews with youth and maternal caregivers using standardised schedules and instruments. These included the Demographics and Questions about Child’s Health schedule, the Family History of Risk Questionnaire, the Child Behaviour Checklist, the Social Experiences Questionnaire and the Survey of Children’s Exposure to Violence. Youth sample comprised 256 participants, with age range from 9 to 18 years, and 65% being female.

**Results:**

Sociodemographic risks were significantly associated with lifetime witnessing violence, victimisation and hearing about violence. Low maternal education was associated with overt peer victimisation and cyber-victimisation. Internalising conditions such as worry and oversensitivity, fear and concentration, youth anxiety and maternal anxiety were also significantly associated with violence exposure and peer victimisation.

**Conclusion:**

Predisposing risks for exposure to violence and victimisation occur in all domains, suggesting that interventions should target these domains to minimise their impact. Co-occurring experience of violence at the personal, proximal and distal levels perpetuate a cyclical loop of violence, intersecting and influencing each other.

**Contribution:**

Risk factors such as anxious attachment, avoidant attachment and anxiety, conceptually often seen as maladaptive outcomes, also serve as predisposing risks for violence exposure.

## Introduction

International studies consistently demonstrate that youth in low socioeconomic status (SES) communities are not only exposed to high rates and distinct types of violence but also show substantive evidence of the adverse effects of violence exposure. Long-term effects of trauma, for example, often persist into adulthood, manifesting as chronic mental health conditions, difficulties in forming healthy relationships and impaired cognitive and emotional development. The prevalence and extent of violence exposure were clearly illustrated in a landmark survey by Verner and Alda,^1^ which showed that violence was a significant risk factor with 73% reporting exposure to and experience of neighbourhood violence, 85% reporting feeling personally unsafe and 13% reporting experiencing violence in the family. Studies continue to show that witnessing violence and exposure to violence and hearing about violence within the home environment or community, impacts the mental health of youth and increases the likelihood of engaging in further violent behaviour. The National Survey of Children’s Exposure to Violence III (NatSCEV) offers comprehensive estimates of victimisation, violence exposure and delinquency patterns among youth and their caregivers in the United States of America.^2^ This survey demonstrates the connections between experiencing violence and various mental health problems such as depression, anger and anxiety.^3^

The linkages between risk and violence exposure, victimisation and other forms of violence feature consistently in the literature as shown in a meta- analysis of longitudinal studies on violence exposure by Castellvi et al.^4^ For example, an American study using a social cognitive processing model to explore associations between exposure to violence, intrusive thoughts and sleep hypothesised that youth develop intrusive thoughts in response to witnessing or directly experiencing violence, which in turn leads to internalising symptoms such as depression and anxiety.^5^

In South Africa, indications are that the youth are increasingly signalling their experiences of stress, trauma and violence as seen in a resurgence of violence in schools and universities, including bullying and victimisation and reported higher levels of suicide and school dropouts.^6^ This is also echoed in international trends, where, for example, the United States National Centre for Health Statistics reports that suicide and homicide rates among adolescents and young adults were on the increase.^7^ There is no doubt that interpersonal violence in South Africa not only dominates injury and mortality rates, loss of healthy life, motor vehicle collisions and alcohol use patterns but also rates significantly high in the global context.^8,9^ Often, however, research findings on the extent, scope and the nature of youths’ experience of violence raise questions because of the inconsistencies and disparities in the reporting of such experiences. Makol et al.^10^ for example, argue that recent research has shown that such reports show significant patterns of both agreements and disagreements between youths and caregivers.

In South Africa, past socioeconomic and structural inequalities, many a legacy of apartheid policies, have created a climate of violence, which continues to perpetuate a cycle of endemic exposure to violence,^11^ as evidenced in high rates of murder, rape, gang violence, gender-based violence, etc. For example, Das-Munshi et al.^12^ in a Cape Town study found that adverse mental health outcomes such as anxiety, depression and posttraumatic stress disorder (PTSD) were more significantly associated with adolescents in historically disadvantaged ethnic groups. Other studies, such as the Western Cape-based study on violence exposure and psychological outcomes,^13^ showed significant associations between exposure to different types of violence (including victimisation and poly-victimisation) and adverse psychological outcomes. Similarly, Hong et al.,^14^ in reviewing numerous international studies on bullying and peer victimisation in adolescents, found that experience and exposure to violence was strongly associated with physical, emotional, behavioural and mental health problems.

Peer victimisation forms part of a complex interrelated system of direct and indirect violence and typically involves the intentional use or threat of physical or psychological violence, which may result in physical injury or psychological harm.^15^ Cole et al.^16^ contend that peer victimisation leads to negative schemas that are related to negative psychological outcomes such as acute stress, trauma symptoms and depression. In a study on risks and resilience among adolescents exposed to community violence in Cape Town, stress and childhood abuse accounted for 33.4% of the variance for PTSD symptoms.^17^ In addition, this study found that black and mixed-race participants were more at risk for exposure to trauma and violence and experiencing PTSD symptoms compared to white participants. An explorative study of 35 risk factors in youth also highlighted the unique burden of risk occurrence in a low socioeconomic community sample in South Africa.^18^ Such studies clearly illustrate that the socioeconomic adversities that characterised marginalised communities during the apartheid regime including low household income, poverty, race, low maternal self-esteem, lack of access to resources, low education and exposure to violence continue to plague vulnerable youth.^19^ The perpetuation of ethnic or racial classification adds to the dynamic of intergenerational transfer of low self-expectation and low aspirations, especially in low SES communities.^20^

One of the damaging effects of apartheid is that the majority, who are black African, have grown up in a society that was marked by political and social violence, further segregated along racial and economic lines. Research on these components has identified several key risk factors including gender, ethnicity, poverty, unemployment, the mental health of a parent, parental educational status, single-parent family, exposure to violence and peer victimisation.^21^ Evans et al.^22^ in their seminal work on cumulative risk and childhood development define low socioeconomic status as being characterised by low household income, low occupational status, generally poor housing quality, low educational status and low resourced communities, noting further that such communities have repeatedly reported poorly resourced schools, poverty and higher levels of community violence. In this study, we look at risk factors and their relation to direct and indirect violence exposure and peer victimisation in a low socioeconomic community youth sample in South Africa.

## Research methods and design

This study is part of a larger collaborative research project on risk and resilience among youth in low socioeconomic communities in Durban, South Africa, titled Project CARE.

It builds on the previous work by Pillay et al.^18^ with the informed consent. The prior work identified and examined 35 significant risk factors in a cohort of low SES youth in Durban, South Africa. The construction of the 35 risk factors is based on data from the interviews and the instruments, informed by the seminal works of Evans et al.^23^ Informed consent was also obtained from all other participants in Project CARE, including the research team involved in the interviews and data management. Using a cross-sectional study design, the 35 risk factors were examined in relation to direct and indirect exposure to violence and peer victimisation.

### Setting

This study was conducted in low socioeconomic communities in Durban, KwaZulu-Natal. They were identified using Evan’s^22^ markers, which include communities with low-cost housing, low household income, poor resources and high crime rates, in combination with government census information and known historical geopolitical demarcations.

### Study participants

The participants (*N* = 324), were youth and their maternal caregivers. Table 1 presents youth demographic information. Youth participants in grade 7 (*n* = 256) and grade 10 (*n* = 68) were recruited from schools within the eThekwini Municipality (Durban) area. Approximately 73.1% (*n* = 237) of the youth live with their biological mothers as the primary maternal caregivers, while 26.9% (*n* = 87) lived with extended maternal caregivers, including stepmothers, grandmothers and elder sisters. Households with more than eight members compromised 19.8% (*n* = 64) of the sample.

**TABLE 1:** Demographic data for youth sample (*N* = 324).

Demographic variable	Frequency	Percentage (%)
**Gender**		
Male	114	35.0
Female	210	65.0
**Youth grade**		
Grade 7	256	79.0
Grade 10	68	21.0
**Racial group**		
Black/African	180	56.0
Mixed race	47	14.0
Indian	75	23.0
White	22	7.0
**Geographical areas**		
Chatsworth	60	18.0
Newlands	101	31.0
Umbilo	22	7.0
Phoenix	59	18.0
Sydenham	40	12.0
Greenwood Park	42	13.0

*Source:* Pillay LG, Pillay BJ, Kliewer W, Sibanda W. An exploration of risk factors in a community sample of low socioeconomic status youth in South Africa. S Afr J Psychol. 2023;53(3):389–402. https://doi.org/10.1177/00812463231186390

Maternal caregivers’ ages ranged from 21 to 75 years (*M* = 41.57, s.d. = 9.86). The maternal caregivers comprised 54.6% (*n* = 177) who were married or co-habiting, while 43.5% (*n* = 141) reported as never married or co-habiting. Most caregivers reported having a grade 9 to grade 12 high school education (70%, *n* = 228) and most reported being either unemployed or unable to work or being full-time housewives or retired (52.8%, *n* = 171). More than a third of the caregivers reported being unemployed or were unable to work. Household income was diverse across the sample, with 142 (43.8%) reporting that their income was unstable and varied from month to month.

### Data collection

Project CARE, from which this study is derived, sampled community schools with grade 7 and grade 10 scholars. This was based on prior studies on risk^22^ and the recognition that this was a significant transitional period for South African scholars. Principals were contacted to participate in the study. Introductory presentations were made on the purposes of the project, the nature of the school’s involvement and added benefits to the school and the community. Learners were addressed and briefed on the project using a standardised script. An information pack, comprising a formal letter of introduction to the caregiver, a reply slip and copies of consent and assent forms, was distributed to youth interested in participating.

The youth and caregivers who submitted their informed consent were contacted, and arrangements were made for interviews by trained research assistants at their homes using structured caregiver and youth interview questionnaires. The youth and caregivers were interviewed separately. All measures and data collected were checked and reviewed by the team of clinical psychologists involved in the research project. This was to ensure validity and reliability of data collected via instruments and to ensure proper data capturing and management.

### Instruments

Various questionnaires were included in the caregiver and child interviews to collect a wide range of data with respect to socioeconomic, psychosocial, psycho-emotional and biomedical factors.

The following questionnaires were part of the Maternal Caregiver Interview:

The **Demographics and Questions about Child’s Health** schedule was developed by the Project CARE research team and was used to collect data on demographic measures, family and parental information and parental assessment of a child’s health.

The **Family History of Risk**, a 22-item questionnaire used to assess the presence or absence of events that could be considered as elevating the risk for maladjustment, was developed by the Programme for Prevention Research in 1999.^24^ The risk constructs included maternal caregiver mental health problems, maternal caregiver suicidal ideation and/or attempts, parental separation or death, child’s exposure to intimate partner violence, etc. An example of an item is ‘Has the child ever experienced the marital separation or divorce of his/her parents?’ Maternal responses are rated as ‘yes’ or ‘no’, and the total number of ‘yes’ responses is summed to create a total risk score. The questionnaire has good construct and face validity.^24^

The maternal caregivers completed the **Child Behaviour Checklist (CBCL)**, which contains a series of 113 items that help assess a child’s behavioural and emotional problems over the 3 months preceding the administration of the questionnaire.^25^ It assesses internalising (i.e. anxious, depressive and overcontrolled) and externalising (i.e. aggressive, hyperactive, noncompliant and undercontrolled) problems with adequate reliability indices, for example, at Cronbach α = 0.81 for anxiety-depression subscales and Cronbach α = 0.77 for the somatisation subscale.

The Youth Interview included the following questionnaires:

The **Social Experiences Questionnaire (SEQ-S)** is a self-report measure of children’s experience of relational victimisation, overt victimisation and cyber-victimisation and is derived from the Problem Behaviour Frequency Scales. Internal consistency ranges from 0.77 to 0.80. Crick et al.^26^ reported a reliability with Cronbach alpha of 0.84 for relational victimisation and Cronbach alpha of 0.64 for cyber-victimisation. A study by Storch et al.^27^ concluded that the scale’s internal consistency was adequate across gender, with the intercorrelations among overt and relational victimisation subscales suggesting that the subscales assess related, but relatively independent constructs of peer victimisation. Cronbach alphas are reported as α = 0.78, α = 0.84 and α = 0.63 for overt victimisation, relational victimisation and cyber-victimisation, respectively.

The **Survey of Children’s Exposure to Violence**, a 40-item survey, was used to measure youth’s exposure to community violence. The survey assesses the frequency that a child has been victimised by, has witnessed or heard about, 20 different forms of violence and violence-related activities in the community. Satisfactory reliability (test-retest, *r* = 0.90; internal consistency, α = 0.85) has been reported.^28^ This survey allows for sub-scores for violence experiences, witnessing and hearing about violence and scores for lifetime and past-year exposure. An index for indirect exposure to violence index is generated by combining violence that is witnessed and heard about. The Cronbach alphas as reported by Richters and Saltzman^29^ read as follows: lifetime victimisation was α = 0.71; past year victimisation was α = 0.61; lifetime witnessing violence was α = 0.89; past year witnessing violence was α = 0.89; lifetime hearing about violence was α = 0.92 and past year hearing about violence was α = 0.93.

### Data analysis

Data analysis was performed using SPSS version 25.^30^ The level of statistical significance was set at *p* < 0.001. A chi-square test was used to determine the associations between the 35 risk factors and exposure to violence and peer victimisation. Violence exposure comprised three sub-measures, namely lifetime exposure to victimisation of violence, lifetime witnessing violence and lifetime hearing about violence. Peer victimisation also included three sub-measures, namely overt victimisation, relational victimisation and cyber-victimisation. Based on the total of three sub-measures, a risk factor that featured ≥ 2 was considered significant. In statistical data analysis, the occurrence of more than two significant subtests in a test of association is significant because it increases the likelihood that the observed associations are not because of chance, thereby enhancing the overall statistical power of the test, making the results more reliable and robust and leading to more confident and reliable conclusions.

### Ethical considerations

Ethical clearance to conduct this study was obtained from the University of KwaZulu-Natal, Biomedical Research Ethics Committee (No. BE052/11). Youth and caregiver participation was voluntary with the choice of withdrawing from the study at any time. All youth and caregiver participants were awarded a shopping voucher as a gesture of appreciation for the time and effort invested in the project.

## Results

Table 1 presents the demographic data for youths participating in the study.

Table 2 presents the 35 risk factors and their associations with community violence exposure and peer victimisation. The results show that among the sociodemographic risk factors, low maternal education was significantly associated with lifetime witnessing violence, X^2^(1, *n* = 317) = 8.410, *p* < 0.004; lifetime victimisation, X^2^(1, *n* = 323) = 5.134, *p* < 0.023 and lifetime hearing about violence, X^2^(1, *n* = 240) = 6.990, *p* < 0.008. Low maternal education was also significantly associated with overt peer victimisation (X^2^[1, *n* = 312] = 6.904, *p* < 0.009) and cyber peer victimisation (X^2^[1, *n* = 322] = 4.565, *p* < 0.033). On the psychosocial risk domain, community violence exposure, victimisation and having a high number of deviant peer relationships were significantly associated with direct and indirect violence exposure and peer victimisation. Similarly, youth worry and oversensitivity, youth fear and concentration, youth and total anxiety and maternal anxiety were significantly associated with measures of violence exposure and measures of peer victimisation. Sixteen (45.71%) of the 35 risk factors were significantly associated with lifetime victimisation, 11 (31.43%) with lifetime witnessing community violence and 12 (34.29%) with lifetime hearing about community violence.

**TABLE 2:** Risk Factors associated with violence exposure and peer victimisation.

Risk factors	Violence exposure				Peer victimisation			
	witlf	victlf	hrdlf	Total %	SEQov	SEQrv	SEQcv	Total %
**Sociodemographic**								
Single-parent household	-	-	-	0	-	-	-	0
Low maternal education	0.004*	0.023*	0.008*	100	0.009*	-	0.033*	66
Low household income	-	0.029*	-	33	-	-	-	0
Race	-	-	-	0	-	0.004*	-	33
Unemployed/handicap	-	-	-	0	-	-	-	0
English nphl parent	-	-	0.006*	33	-	-	-	0
English nphl youth	-	-	0.004*	33	-	-	-	0
**Psychosocial**								
Household size	-	-	-	0	-	0.038*	-	33
Household quality	-	-	-	0	-	-	-	0
Family stress	-	-	-	0	-	-	-	0
Maternal mental health problems	-	-	-	0	-	-	-	0
Mental illness/suicidal ideations	-	0.024*	-	33	-	-	-	0
High community violence exposure	0.000**	0.000**	0.000**	100	0.000**	0.000**	0.000**	100
High peer victimisation	0.000**	0.000**	0.015*	100	0.005	0.037*	0.000**	100
High number of deviant peers	0.000**	0.000**	0.000**	100	0.000**	0.000**	0.000**	100
**Psycho-emotional**								
Low parent knowledge of youth – youth rated	0.000**	0.000**	-	66	-	-	-	0
Low parent knowledge of youth – parent rated	-	-	-	0	0.030	-	-	33
Avoidant attachment	0.013*	0.001**	-	66	-	-	0.042*	33
Anxious attachment	0.005*	0.005*	-	66	-	-	-	0
Low mat. self-esteem	-	-	-	0	-	-	-	0
Low dyadic satisfaction	-	-	-	0	-	-	-	0
Low emotion regulation, high lability P	-	-	-	0	-	-	-	0
Low anger regulation Y	-	-	-	0	0.010*	0.048*	-	66
Youth depression	-	-	-	0	-	-	-	0
Youth somatisation	-	0.002*	0.047*	66	0.002*	0.012*	-	66
Youth physiological anxiety	-	-	-	-	-	-	-	-
Youth worry and sensitivity	0.001**	0.001**	0.005*	100	0.000**	0.000**	-	66
Youth fear and concentration	-	0.008*	0.037*	66	0.005*	0.001**	0.040*	100
Youth total anxiety	0.001**	0.001**	0.003*	100	0.000**	0.000**	-	66
Maternal somatisation	-	-	-	0	-	-	-	0
Maternal depression	-	0.031*	0.007*	66	-	-	-	0
Maternal anxiety	0.036*	0.001**	0.006*	100	-	-	-	0
Maternal hostility	-	0.030*	-	33	-	-	-	0
**Biomedical**								
Youth – chronic handicap	-	-	-	0	-	-	-	0
Youth poor health – parent rated	0.017*	-	-	33	0.006	0.001**	-	66
**Total**	**11**	**16**	**12**	-	**11**	**11**	**6**	-
**Total percentage (%)**	**31.43**	**45.71**	**34.29**	-	**31.43**	**31.43**	**17.14**	-

Witlf, lifetime witnessing violence; Victlf, lifetime victimisation; hrdslf, lifetime hearing about violence; SEQov, Social Experiences Questionnaire – overt victimisation; SEQrv, Social Experiences Questionnaire – relational victimisation; SEQcv, Social Experiences Questionnaire – cyber-victimisation; nphl, nphl-not primary home language.

* , *p* < 0.05;

** , *p* < 0.0.

## Discussion

This study investigated the relationship between 35 risk factors and direct and indirect violence exposure and peer victimisation in a diverse low socioeconomic community sample. The negative impact of exposure to violence on youth development, mental health and academic achievements has been well documented.^5,31,32,33^ In the literature on violence exposure and victimisation, much of the focus is on the maladaptive psychological outcomes, especially in terms of internalising and externalising disorders and interventions.^13^ However, the issue of ‘the risk factors that predispose youth to direct and indirect exposure to violence and peer victimisation’ has received little or no attention. This is especially crucial not only in intervention and treatment approaches but also to appropriately inform policies and broad-based preventative strategies. A previous study by Pillay et al.^18^ on risk factors, which explored the unique burden of risks on youth in a community sample, showed that risk factors such as low household income, race, English as second language, low maternal self-esteem and low dyadic satisfaction were not only significant but also highlighted the linkages of these risk factors to different experiences of violence including displays of aggression, bullying, antisocial behaviour, delinquency and vandalism. Our study sought to explore these ‘predisposing’ risk factors to add to the literature on violence exposure and treatment approaches in South Africa.

Low maternal education emerged as a high sociodemographic risk for youth, associated with lifetime witnessing violence, hearing about violence and both overt and cyber peer victimisation. Mothers with low education may not view indirect exposure to violence and victimisation as being critical to a child’s development as direct experience of violence and may therefore be less disposed to attending to the potential negative impact. Additionally, mothers with lower education may suffer greater helplessness in dealing with violence and may further engage a ‘normalisation’ defence strategy. Mothers with low education may also lack the self-confidence and may experience more challenges in accessing resources and institutional assistance. While low household income (alluding to poverty) only features as a significant risk for lifetime victimisation, the combination with poor maternal education and environmental deprivation creates a cumulative disadvantage, where each risk reinforces the negative impact of the other. For instance, a mother with limited education may have fewer job opportunities, leading to poverty, which in turn exacerbates the challenges posed by an impoverished environment. A US-based study, for example, found high rates of past-year exposure to violence for youth residing in high-disadvantage neighbourhoods.^34^

While maternal emotional dysregulation did not emerge as significant, it is nonetheless a critical covariate of low maternal education, which places children at risk for psychological and behavioural problems^35^ and also negatively impact on parents’ ability to model appropriate coping behaviours to optimally support their children in avoiding and coping with violence and victimisation. While there has been a substantial call for intervention at the individual and community level, for youth exposed to violence,^36^ the risk profile of this study suggests that given the ubiquitous nature of violence exposure, maternal caregivers must also be empowered with information on violence and be given priority, especially with respect to access to resources. It would be prudent for institutions to also reach directly into households and neighbourhoods rather than waiting for ‘disempowered caregivers’ to reach out. While maternal mental health is a strong determinant in child development,^37^ our study also found maternal anxiety and depression to be significant risk factors for exposure to violence. It is hypothesised that parents who suffer depression and anxiety may be emotionally detached and may suffer reduced capacity for daily activities. Children in such situations may experience more stress, engage in self-blame and perceive the parent as unavailable in providing support to cope with their experiences of violence or victimisation. Interventions with youth must necessarily include working with parents, focusing on conditions such as maternal depression and anxiety and building the parent–child relationship.

As expected, exposure to community violence is a significant risk factor for victimisation, and conversely, victimisation increases the likelihood of exposure to violence. The experience of violence is rarely isolated, often perpetuating a cyclical loop of violence, exposure and victimisation. Leoschut et al.^38^ in examining the frequency and predictors of poly-victimisation in a South Africa sample found that co-occurring forms of violence intersect and influence each other. It is crucial to understand these cyclical forms of violence at personal, proximal and distal levels to develop appropriate interventions for vulnerable youth.

Our study also shows that factors traditionally seen as maladaptive outcomes additionally serve as predisposing risk factors for violence exposure. For instance, while youth with numerous deviant peers may exhibit outcomes such as anxious attachment, avoidant attachment, anxiety, fear and low concentration,^4,39^ these traits, in turn, predispose them to increased exposure to violence and victimisation. This may occur through various mechanisms. For example: (1) youth with high levels of anxiety or fear may have impaired judgment and decision-making abilities, making them more likely to engage in risky behaviours or associate with deviant peers; (2) anxiety and fear can heighten stress responses, making youth more reactive to threats, leading to behaviours that provoke or attract violence; (3) avoidant attachment can result in reluctance to seek help from supportive adults or peers, leaving youth more isolated and vulnerable to victimisation; (4) peers and perpetrators of violence may perceive anxious or fearful youth as easy targets, increasing the likelihood of victimisation; and (5) youth with these traits may use maladaptive coping strategies such as substance abuse or aggression, which can further expose them to violent environments. Together these factors create a cycle where maladaptive traits increase the likelihood of encountering violence, which in turn can reinforce and exacerbate these traits.

## Conclusion

Our study clearly shows that the risk factors associated with exposure to violence and peer victimisation occur on all levels, including the sociodemographic, psychosocial, psycho-emotional and biomedical clusters. While studies often recommend macrolevel interventions such as community empowerment, legislation and policy development,^40^ our findings suggest that interventions should also be designed around the different clusters in which predisposing risk occurs. For example, notwithstanding the findings by Dray et al.^41^ that school-based prevention programmes showed little effectiveness in prevention of mental health problems in youth, interventions may adopt a more inclusive and holistic approach, targeting not only the youth but also parents, schools and the community. The goal of these multifaceted interventions is to minimise predisposing risks by developing strategies to reduce exposure to violence and victimisation and to build resilience and fortitude among youth, parents and neighbourhoods.^42^ Our study clearly shows the complex interaction between risks, violence exposure and peer victimisation and demonstrates that these interactions occur on different levels and in different clusters.

Although this study presented an inclusive taxonomy of risk factors and its associations with violence exposure and victimisation, in a representative sample of youth in KwaZulu-Natal, certain limitations are noted. Initially, the use of a cross-sectional design did not yield longitudinal data, especially to track changes in patterns of occurring risks and associations over time. This study also utilised self-report inventories, which could be subject to inaccurate understanding of constructs, poor recall and misrepresentation. Despite the potential impact of low maternal education, trained research assistants mitigated these issues, enhancing the reliability and validity of written responses. The addition of qualitative clinical interviews may be useful in increasing the veracity of the data. Qualitative interviews allow nuanced information about thoughts, feelings and behaviours; provide a deeper understanding of context; can reveal underlying motivations and allow for clarification of ambiguous or incomplete responses.

While the study reflects the overall demographics of the race groups in South Africa, it would be useful if more equitable numbers of participants from the different race groups were included in the sample. In addition, this study did not include gender-specific factors and associations. Given the growing incidences of reported gender-based violence, it is imperative that future research must necessarily explore gender as a risk and its association with violence exposure. This would endear such studies with critical community and societal relevance and would also consider a fuller spectrum of the experience of violence in our communities. Nonetheless, this study contributes uniquely to the literature on risk factors and to the understanding of the associations between risk factors and violence exposure and peer victimisation among youth in low socioeconomic communities in South Africa.
